# Activity of *Ajuga iva* Extracts Against the African Cotton Leafworm *Spodoptera littoralis*

**DOI:** 10.3390/insects11110726

**Published:** 2020-10-23

**Authors:** Leena Taha-Salaime, Galina Lebedev, Jackline Abo-Nassar, Sally Marzouk, Moshe Inbar, Murad Ghanim, Radi Aly

**Affiliations:** 1 Department of Evolutionary and Environmental Biology, The Faculty of Natural Science, University of Haifa, Haifa 3498838, Israel; leena.ta.salaime@gmail.com (L.T.-S.); minbar@research.haifa.ac.il (M.I.); 2 Department of Plant Pathology and Weeds Research, Newe Ya’ar Research Center, Agricultural Research Organization, Ramat Yishay 30095, Israel; jackline@volcani.agri.gov.il (J.A.-N.); sally.marzouk91@gmail.com (S.M.); 3 Department of Entomology, Agricultural Research Organization, The Volcani Center, Rishon LeTsiyon 7528809, Israel; galinal@volcani.agri.gov.il (G.L.); ghanim@volcani.agri.gov.il (M.G.)

**Keywords:** *Ajuga iva*, clerodane, pest control, phytoecdysteroid, *Spodoptera littoralis*

## Abstract

**Simple Summary:**

Pest insects cause tremendous damage and losses to global agriculture, and their control relies mainly on chemical insecticides, which are greatly harmful for human health and the environment. Many *Ajuga* plant species have secondary metabolites such as phytoecdysteroids (analogues of insect steroid hormones—ecdysteroid) that control insect development and reproduction. In this study, the effect of ingestion of *Ajuga* *iva* phytoecdysteroid plant extract on the growth and development of the African cotton leafworm *Spodoptera littoralis* was carried out. Our results clearly showed the susceptibility of *S. littoralis* to phytoecdysteroid ingestion. Crude leaf extracts and fractionated phytoecdysteroids significantly increased mortality of first-instar *S. littoralis* by up to 87%. Third-instar larval weight gain decreased significantly by 52%, and crude leaf extract (250 ppm) reduced gut size, with relocation of nuclei and abnormal actin-filament organization compared to the controls. In conclusion, the use of phytoecdysteroids against pest insects is not an alternative for chemical insecticides, but they could have an important role in integrated pest management strategies for controlling *S. littoralis* and possibly other lepidopterans.

**Abstract:**

Control of the crop pest African cotton leafworm, *Spodoptera littoralis* (Boisduval), by chemical insecticides has led to serious resistance problems. *Ajuga* plants contain phytoecdysteroids (arthropod steroid hormone analogs regulating metamorphosis) and clerodanes (diterpenoids exhibiting antifeedant activity). We analyzed these compounds in leaf extracts of the Israeli *Ajuga iva* L. by liquid chromatography time-of-flight mass spectrometry (LC-TOF-MS) and thin-layer chromatography (TLC), and their efficiency at reducing *S.* *littoralis* fitness. First and third instars of *S. littoralis* were fed castor bean leaves (*Ricinus communis*) smeared with an aqueous suspension of dried methanolic crude extract of *A. iva* phytoecdysteroids and clerodanes. Mortality, larval weight gain, relative growth rate and survival were compared to feeding on control leaves. We used ‘4’,6-diamidino-2-phenylindole (DAPI, a fluorescent stain) and phalloidin staining to localize *A. iva* crude leaf extract activity in the insect gut. *Ajuga iva* crude leaf extract (50, 100 and 250 µg/µL) significantly increased mortality of first-instar *S. littoralis* (36%, 70%, and 87%, respectively) compared to controls (6%). Third-instar larval weight gain decreased significantly (by 52%, 44% and 30%, respectively), as did relative growth rate (−0.05 g/g per day compared to the relevant controls), ultimately resulting in few survivors. Crude leaf extract (250 µg/µL) reduced gut size, with relocation of nuclei and abnormal actin-filament organization. *Ajug iva* extract has potential for alternative, environmentally safe insect-pest control.

## 1. Introduction

The African cotton leafworm *Spodoptera littoralis* (Boisduval) is considered one of the most serious pests of cotton, maize, rice, alfalfa, potato, tomato, ornamentals, and orchard trees [1]. It feeds year-round on the leaves of numerous old- and new-world plant species [2]. Today, insect pests are mainly controlled by insecticides, which constitute a risk to human health and the environment [3]. Many insecticides have been derived from plant sources, and some, such as alkaloids, terpenoids, phenols, and steroids, exhibit very high toxicity against a variety of agricultural pests. In this study, we examined the potential use of phytoecdysteroids and clerodanes extracted from *Ajuga* (Lamiaceae) plants to control *S. littoralis*. 

Phytoecdysteroids are plant-produced steroids that are analogs of the steroid hormones that control molting and metamorphosis in arthropods [4]. Phytoecdysteroids are present in 5–6% of plant species [5], generally at higher concentrations than those typically found in arthropods [6]. Most of them possess a cholest-7-en-6-one carbon skeleton (C27) and are synthesized from phytosterols in the cytosol through the mevalonic acid pathway [4]. They can mimic insect 20-hydroxyecdysteroid, bind insect ecdysone receptors, and elicit the same responses [7]. Phytoecdysteroids may cause abnormal larval development, feeding deterrence, and ultimately, death [7]. Ecdysteroids are not toxic to mammals, because their structure is quite different from mammalian steroids, and they are not expected to bind to vertebrate steroid receptors [8]. 

Ecdysone, a natural molting hormone of insects derived from enzymatic modification of cholesterol by P450 enzymes [9], controls developmental events by changing the levels of other ecdysteroids [10]. The ecdysone receptor is a nuclear receptor (a ligand-activated transcription factor) that binds to and is activated by ecdysteroids. In *Manduca sexta* larvae, 20-hydroxyecdysone is primarily produced in the prothoracic gland, gut, and fat bodies [11] from dietary cholesterol and acts through the ecdysone receptor [12]. In addition, the ecdysone receptor controls development and contributes to other processes (such as reproduction) [13] and to interactions between the cytoskeleton (the effector of cell movement and changes in cell shape) and changes in the distribution of actin staining and microfilaments [14]. 

Discovery of the same molecules (phytoecdysteroids) in several plant species suggests that they may be effective against insect herbivores by acting as antifeedants and/or disrupting the insects’ endogenous endocrine levels [4,15]. Kubo [16] reported that an extract of *Ajuga remota*, containing 20-hydroxyecdysone and cyasterone, added to the diet of *Bombyx mori* inhibited ecdysis, resulting in larval retention of the exuvial head capsule and the insect’s death. Similarly, larvae of the greenhouse whitefly (*Trialeurodes vaporariorum*) exhibited 100% mortality when fed on *Ajuga reptans* plants. High levels of the three major phytoecdysteroids, 20-hydroxyecdysone (ecdysterone), makisterone A and cyasterone, have been found in several plants, including *Ajuga* [4,17,18,19,20,21,22,23,24,25,26], quinoa and spinach [4,27]. An extract of 20-hydroxyecdysone and cyasterone from *Ajuga iva* L. showed high activity against *Oligonychus perseae* [28,29]; a dose of 5 μg/mL of pure extracted *A. iva* ecdysterone significantly reduced fecundity, fertility and survival of this pest, while commercial 20-hydroxyecdysone at the same dose had lesser effects [29]. 

In addition to phytoecdysteroids, species of the genus *Ajuga* also contain the bioactive compounds clerodane diterpenes (including clerodanes) and iridoid glycosides [30]. Clerodanes (diterpenoids) are a large group of C20 terpene compounds derived from geranylgeranyl diphosphate and biosynthesized through the deoxyxylulose phosphate pathway in the cytoplasm, mostly in the leaves and stems of the Lamiaceae and Asteraceae families [31]. Clerodin was originally isolated from *Clerodendrum infortunatum* (Lamiaceae), and has potential as a natural pesticide due to its insect repellent activities [20,32,33,34]. Koul [33] showed that the most active compounds, dihydroclerodin and clerodin hemiacetal from *Caryopteris divaricata*, exhibit 100% antifeedant activity at 50 ppm. These clerodanes were deadly to *Spodoptera litura*. 

We previously identified and quantified high contents of three phytoecdysteroids and two clerodanes in *A. iva* growing in Israel [26]. We hypothesized that a crude extract of *A. iva* leaves that includes the three phytoecdysteroids (20-hydroxyecdysone, makisterone A and cyasterone), which specifically interfere by controlling molting and are responsible for metamorphosis and antifeedant activities in insects, might be a promising pest-control agent. We evaluated the efficacy of *A. iva* extracts (containing phytoecdysteroids and clerodanes) in reducing the damage caused by *S. littoralis* by addressing the following questions: Does *A. iva* crude leaf extract affect *S. littoralis*? Do phytoecdysteroids isolated from the crude leaf extract and commercial standards have different effects on the larvae? Do phytoecdysteroids have a direct effect on the larvae’s gut? 

## 2. Materials and Methods

### 2.1. Plants and Insects

*Ajuga iva* plants were collected in April 2014 from a wild population in the Negev, southern Israel, and then cultivated and acclimated in an open field at Newe Ya’ar Research Center (32°43′02.5284″ N, 35°17′49.3008″ E). Young and mature leaves and stems of fresh plants were collected after blooming (July–November) and oven-dried at 55 °C for 3–4 days, then homogenized to a fine powder prior to extraction. The first and third instars of *S. littoralis* used for the bioassays were from Murad Ghanim’s laboratory, Department of Entomology, Agricultural Research Organization (African cotton leafworm colony) reared on castor bean leaves under laboratory conditions (25 ± 1 °C, 40% relative humidity with a 12–12 h light–dark cycle). 

### 2.2. Extraction and Purification of Phytoecdysteroids

*Ajuga iva* crude extracts were prepared according to our recently published procedure [27]. Leaf and stem powder were pooled (24 g) and soaked in 240 mL of 100% MeOH, sealed and homogenized with shaking (2500 rpm) for 1 h. The extract was then centrifuged (12,000× *g*) for 10 min, filtered and concentrated under vacuum. The final filtered methanol solution was analyzed by liquid chromatography–time of flight-mass spectrometry (LC–TOF-MS) (Newe Ya’ar Research Center, Agricultural Research Organization, Ramat Yishay, Israel) and dried in a chemical vaporizer for 5 days. For purification of phytoecdysteroids from the crude extract, leaf, and stem powder (100 g) was soaked in 300 mL methanol and homogenized. The filtered extract was vacuum-concentrated and treated with H_2_O to give 30% aqueous methanol. This solution was extracted as previously described [29].

### 2.3. Identification of Phytoecdysteroids and Clerodanes 

LC–TOF-MS analysis was used to identify and confirm the presence of phytoecdysteroids and clerodanes in three concentrations of *A. iva* crude leaf extract (50, 100 and 250 µg/µL). We analyzed the profile of phytoecdysteroids and clerodanes in the *A. iva* crude leaf extract before each test for biological activity. Extracts of the plant material (1 µL) were injected into an Agilent 1290 Infinity Series liquid chromatograph coupled with an Agilent 1290 Infinity DAD and Agilent 6224 Accurate Mass TOF mass spectrometer (Agilent Technologies, Santa Clara, CA, USA) [9]. Thin-layer chromatography (TLC) (Sigma-Aldrich, Israel, Product Number: 1.05553.0001, Rehovot 7670603, Israel) was used to separate the components into well-defined spots. The crude leaf extract, the pure isolated compounds (20-hydroxyecdysone (ecdysterone), makisterone A and cyasterone) and a commercial ecdysterone sample were applied to silica gel GF-254 plates (0.25 mm; 20 × 20 cm), as described in Aly et al. [29].

### 2.4. Biological Activity of A. iva Crude Leaf Extract against S. littoralis

To assess the effects of *A. iva* on *S. littoralis*, mature castor bean leaves were smeared, using a paint brush, with aqueous *A. iva* crude leaf extract (24 g of dried pooled leaves and stems dissolved in 240 mL MeOH, 1:10) and Tween 20 (1.5 mg). The leaves were dried in a chemical hood for 2 h. Then, 10 first-instar *S. littoralis* were placed on 1 treated castor bean leaf in a petri dish and allowed to feed for 3 days in a climate-controlled room at 25 °C. Control leaves were similarly smeared with double-distilled water (ddH_2_O) and Tween 20. The larvae were exposed to three concentrations of crude leaf extract (50, 100 and 250 µg/µL), one concentration per treatment. After preparing the *A. iva* crude leaf extract, the methanolic extract was dried in a chemical hood; 1.2 g dried extract powder was dissolved in 4.8 mL ddH_2_O and Tween 20 (0.5 mg/mL) for the 250 µg/µL concentration; 0.83 mL of the high concentration (250 µg/µL) extract was dissolved in 3 mL ddH_2_O to obtain the 100 µg/µL concentration; and 0.67 mL of the 100 µg/µL solution was dissolved in 3 mL ddH_2_O to obtain the 50 µg/µL concentration. At the end of the experiment, larval mortality was compared to that of controls. Data in this experiment represent the results of 11 replicates (10 larvae/replicate). Differences are reported as percent mortality of first-instar larvae after feeding on the three concentrations of *A. iva* crude leaf extract using a *t*-test (*p* ≤ 0.05), and significance was determined by *t*-test.

In another experiment, for the third instars, 1 or 10 larvae were fed on 1 treated castor bean leaf for 4 or 8 days in a climate-controlled room at 25 ± 1 °C (more freshly treated leaves were provided after 4 days of feeding to avoid feeding on decayed leaves). Four different treatments were tested, where larvae were fed on castor bean leaves treated with the following: (1) 250 µg/µL *A. iva* crude leaf extract for 4 days, with a freshly treated leaf for 4 more days; (2) the same treatment as (1) with 250 µg/µL of a fractionated mixture of three phytoecdysteroids from *A. iva* leaf extract; (3) *A. iva* crude leaf extract for 4 days, and then a control castor bean leaf smeared with double-distilled water (ddH_2_O) and Tween 20 for the next 4 days; (4) a control castor bean leaf for 4 days and then *A. iva* crude leaf extract for the next 4 days. In parallel, control leaves were smeared with ddH_2_O and Tween 20 for 8 days. We recorded the different reactions of *S. littoralis* in all treatments after 4 days, and whether larvae could recover if provided a control castor bean leaf (after first being fed on a treated leaf) or were adversely affected when provided a treated castor bean leaf after 4 days of feeding on a control leaf. In another experiment, third-instar larvae were exposed to three concentrations of *A. iva* crude leaf extract (50, 100 and 250 µg/µL) for 4 days. At the end of the experiment, we recorded larval survival and relative growth rate (RGR) as (ln *W*_2_ − ln *W*_1_)/(*t*_2_ − *t*_1_), where *W*_1_ and *W*_2_ are weights at times *t*_1_ and *t*_2_ (*n* = 20 replicates). In each treatment, pupation rate was evaluated for an additional 15 days. The RGRs were compared using a mixed-model ANOVA (repeated measures ANOVA until day 4 and two-way ANOVA from day 4 to the end of the experiment). Survival rates were analyzed by the Friedman test, because the data for larval survival did not follow a normal distribution. Data for larval weight gain (LWG) are the result of 10 replicates (10 larvae in each replicate). The LWGs were compared using a repeated measures ANOVA. Statistical significance was reported at *p* < 0.05. Error bars in all graphs represent the standard error of the mean (SEM), and significance is indicated in each experiment. All statistical analyses were performed with IBM SPSS software v.20 for Windows (IBM, Armonk, NY, USA). 

### 2.5. Effect of A. iva Crude Leaf Extract and Purified Phytoecdysteroid Mixture on Larval Gut of S. littoralis 

To examine the gut morphology of *S. littoralis*, we used DAPI, a fluorescent stain and phalloidin staining. Larvae fed on castor bean leaves treated with *A. iva* crude leaf extract or controls (leaves treated with water) were tested after 7 days of treatment. Guts were dissected in phosphate buffered saline (1× PBS), then fixed in 4% paraformaldehyde in 1× PBS for 30 min, washed in 0.1% (*w*/*v*) Triton X-100 (Sigma-Aldrich, CAS-Number: 9002-93-1, Rehovot 7670603, Israel) for 30 min, washed three times in PBS Tween-20 (PBST) (https://www.usbio.net/protocols/phosphate-buffered-saline-Tween-20) [35], incubated in 0.1% (*w*/*v*) phalloidin in PBST for 30 min, washed three times with PBST, and mounted whole in hybridization buffer (20 mM Tris-HCl pH 8.0, 0.9 M NaCl, 0.01% *w*/*v* sodium dodecyl sulfate, 30% *v*/*v* formamide) with 0.1% (*w*/*v*) DAPI, a fluorescent stain. Changes in actin fibers and nuclei were visualized under a confocal microscope (Leica SP8 (Agricultural Research Organization, The Microscopy Unit of the Volcani center, Rishon LeTsiyon) and Olympus IX 81 (Agricultural Research Organization, The Microscopy Unit of the Volcani center, Rishon LeTsiyon).

## 3. Results 

### 3.1. Identification of Natural Phytoecdysteroids from A. iva by TLC Analysis 

**Figure 1:**
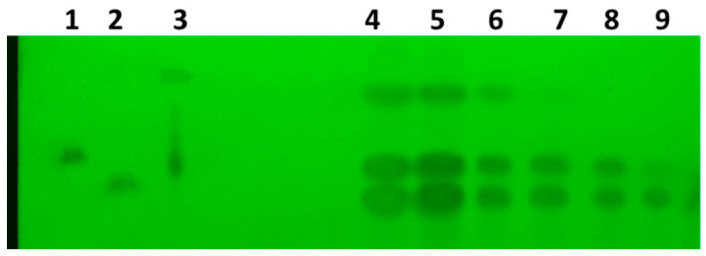
Identification of *A. iva* phytoecdysteroids by Thin-layer chromatography (TLC). TLC plate shows the separation of three phytoecdysteroids (20-hydroxyecdysone, makisterone A and cyasterone) (lanes 4–6), and commercial ecdysterone standards (lanes 1–3): makisterone (**1**), 20-hydroxyecdysone (**2**), and cyasterone (**3**). Fractions 7–9 show the presence of only 20-hydroxyecdysone and cyasterone. Fractions 4–6 were used in the bioassays.

*Ajuga iva* crude leaf extract was subjected to flash chromatography on a silica gel (TLC), yielding three individual isolated compounds (20-hydroxyecdysone, makisterone A and cyasterone). The retention factor (Rf) values, i.e., the distance migrated over the total distance covered by the solvent, of the phytoecdysteroid spots were similar to those of the respective commercial ecdysteroids (Figure 1). 

### 3.2. Effect of A. iva Crude Leaf Extract on First-Instar Larval Survival 

**Figure 2:**
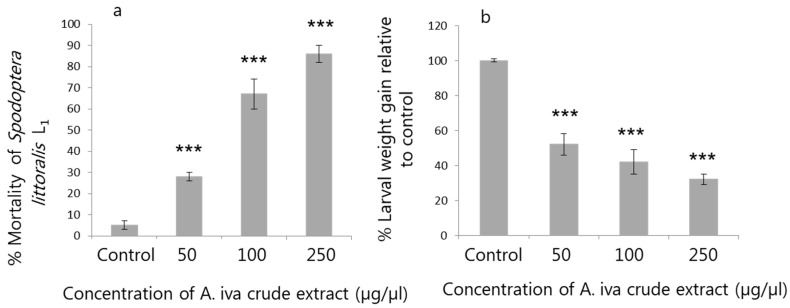
Effect of different concentrations of *A. iva* crude leaf extract on *S. littoralis* first-instar (L_1_) larval (*n* = 110) mortality (mean ± SEM) (**a**), and larval weight gain (%) (mean ± SEM) of *S. littoralis* third-instar (L_3_) larvae (**b**). Asterisks (***) indicate significant difference (*p* ≤ 0.05) by *t*-test (t_108_ = 6.105, 4.308, and 3.220 for 50, 100 and 250 µg/µL, respectively); *p <* 0.001 for all treatments, Levene’s test *p* = 0.326 (**a**), and by repeated measures ANOVA (F_3.104_ =20.334, 17.246, and 13.007 for 50, 100, and 250 µg/µL, respectively); *p <* 0.001, Mauchly’s test *p* = 0.152 (**b**) between treatments and the control.

First-instar *S. littoralis* showed a significant increase in mortality (25, 65 and 85%) after feeding on the three concentrations of *A. iva* crude leaf extract (50, 100 and 250 µg/µL, respectively), compared to the control (treated with water, 5%) (Figure 2a).

### 3.3. Effect of A. iva Crude Leaf Extract on Third-Instar Larval Survival and Development 

Third-instar *S. littoralis* fed on crude leaf extract (50, 100 and 250 µg/µL) showed reduced LWG (F_3.104_ = 20.334, 17.246, and 13.007, respectively, *p <* 0.001; Figure 2b) compared to the control. 

**Figure 3:**
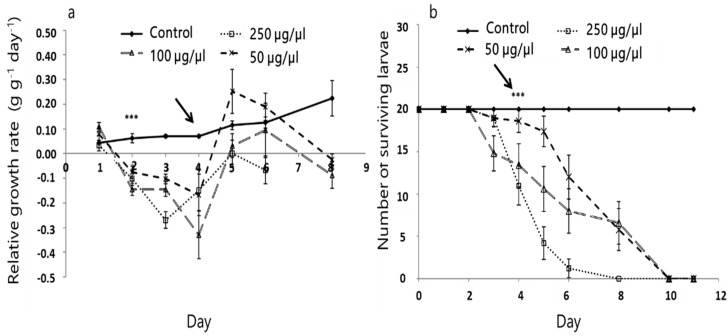
Effect of different concentrations of *A. iva* crude leaf extract on *S. littoralis* third-instar larval relative growth rate (mean ± SEM) (**a**) and survival (**b**); *n* = 20. (***) Asterisks indicate significant difference (*p* ≤ 0.05) between treatments and the control.

All concentrations of crude leaf extract significantly decreased (*p* < 0.001) larval RGR compared to the normally developing larvae on the control diet (Figure 3a, arrow). Reduced RGR was recorded as early as 2 days into the experiment. Interestingly, we found that with the highest concentration of crude leaf extract, RGR decreased by 0.05 and 0.20 g/g per day on days 6 and 8, respectively, compared to the control (F_3.16, 18_ = 12.641, *p <* 0.001; Figure 3a). All concentrations of *A. iva* crude leaf extract significantly reduced third-instar larval survival after 11 days (*X*^2^_3_ = 6.221, *p* = 0.038; Figure 3b). Whereas all larvae survived on the control leaves, the effect of the crude extract was apparent after 3 days. In fact, none of the treated larvae survived more than 8 days for the highest concentration of crude leaf extract and 10 days for the other concentrations (Figure 3b).

**Figure 4:**
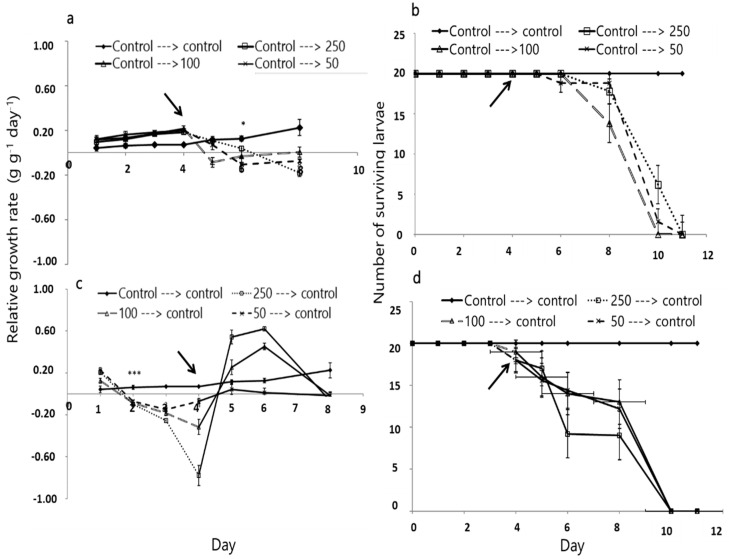
Effect of *A. iva* crude leaf extract on *S. littoralis* third-instar larval relative growth rate (mean ± SEM) and survival. Larval relative growth rate (**a**) and survival (**b**) when fed on control leaf (treated with water and Tween) until day 4, and then fed on leaves treated with crude leaf extract at three concentrations until the end of the experiment (**a**) (control treated with DDW and Tween for all the 8 days). Relative growth rate (**c**) and survival (**d**) of the larvae after feeding on crude leaf extract at three concentrations until day 4 and then control leaves until the end of the experiment (control treated with DDW and Tween for all the 8 days); *n* = 20. (***) Asterisks indicate significant difference (*p* ≤ 0.05) between treatments and the control. Arrow points to day 4.

In addition, when *S. littoralis* were first fed on control leaves for 4 days and then on leaves treated with *A. iva* crude leaf extract for an additional 4 days, their RGR was affected by feeding on the crude leaf extract after day 5 of the experiment (F_3.16, 18_ = 7.310, *p <* 0.001; Figure 4a), and continued to decrease until the end of the experiment, with no surviving larvae (*X*^2^_3_ = 9.282, *p* = 0.021; Figure 4b).

The same result was obtained when the order of the treatments was reversed (Figure 4c,d). When the larvae were first fed on leaves treated with *A. iva* crude extract for 4 days and then fed on control leaves (for an additional 4 days), a significant decrease in RGR was obtained on days 2–4 (F_3.16, 18_ = 4.595, 3.608, and 8.113, *p* = 0.034, 0.02, and 0.001 for 50, 100 and 250 µg/µL crude leaf extract, respectively; Figure 4c).

**Figure 5:**
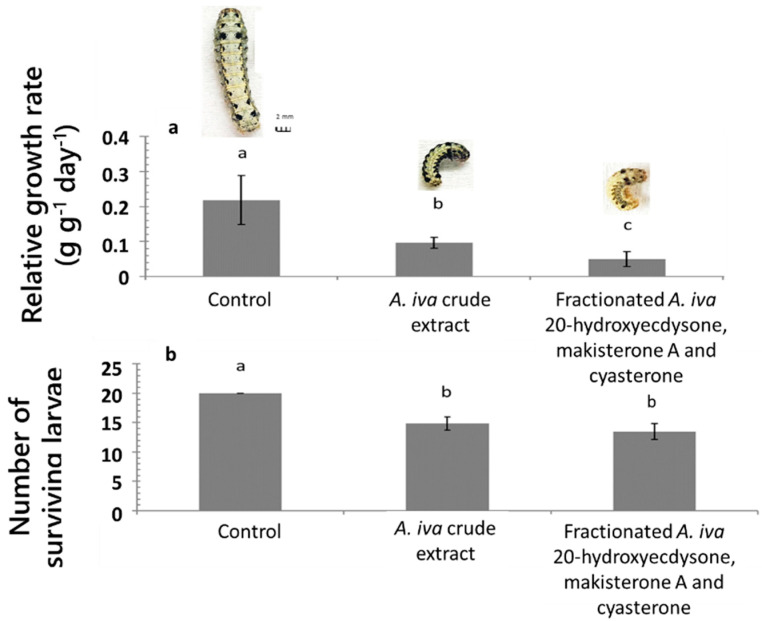
Effect of *A. iva* crude leaf extract (250 µg/µL), and of the three fractionated and purified phytoecdysteroids (250 µg/µL) on *S. littoralis* third-instar larval relative growth rate (mean ± SEM) (**a**) and survival (**b**). The phytoecdysteroid fraction contained 20-hydroxyecdysone, makisterone A and cyasterone; *n* = 20. Development of *S. littoralis* shown above the columns after 4 days feeding on control leaves, or leaves treated with 250 µg/µL *A. iva* crude leaf extract or 250 µg/µL of the three fractionated phytoecdysteroids. Different letters above columns indicate significant difference (*p* ≤ 0.05) between treatments and the control.

Moreover, castor bean leaves treated with 250 µg/µL of the mixture of the three fractionated and purified phytoecdysteroids from the *A. iva* crude leaf extract significantly reduced RGR (F_2.20, 18_ = 6.172, *p* = 0.001)> compared to the control (Figure 5a). A few larvae survived on the leaves treated with purified phytoecdysteroid fraction (*X*^2^_3_ = 11.305, *p* = 0.04) (Figure 5b).

Overall, larvae fed on castor bean leaves treated with 250 µg/µL *A. iva* crude leaf extract or 250 µg/µL of the phytoecdysteroid mixture lost weight, stopped growing and ultimately died (Figure 5a, larvae depicted above columns).

### 3.4. Effect of A. iva Crude Leaf Extract and Purified Phytoecdysteroid Mixture on Larval Gut of S. littoralis 

**Figure 6:**
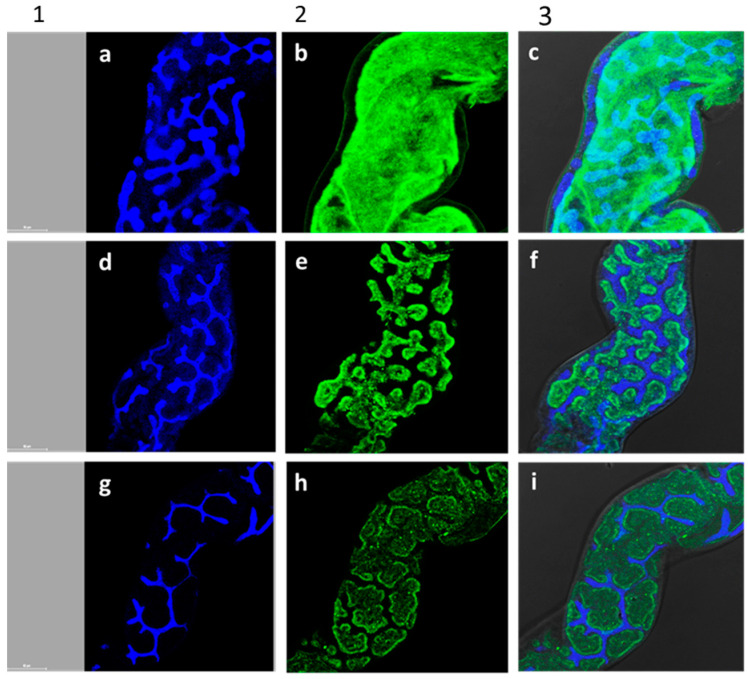
Gut morphology of *S. littoralis* third-instar larvae after feeding on treated or non-treated leaves: control castor bean leaves treated with water (**a**–**c**), leaves treated with *A. iva* crude leaf extract (250 µg/µL) (**d**–**f**), and leaves treated with 250 µg/µL of three fractionated and purified phytoecdysteroids from *A. iva* leaf extract (20-hydroxyecdysone, makisterone A and cyasterone) (**g**–**i**) for 8 days (60 µm, respectively). Blue: DAPI, a fluorescent stain, staining of the nuclei under dark field (1); green: phalloidin staining of actin filaments under dark field (2); double DAPI, a fluorescent stain, staining of the nuclei and phalloidin staining of actin filaments under dark field (3).

Larval guts were stained with phalloidin, an actin-specific marker that binds to the interface between adjacent actin monomers in the F-actin polymer, and with DAPI, a fluorescent stain, which stains the nuclei. Larvae feeding on 250 µg/µL *A. iva* crude leaf extract for 8 days had smaller nuclei with an abnormal shape—the nuclei moved to the edges of the cell and were thinner than normal (Figure 6d–f). Phalloidin staining showed normal actin-filament organization in the control treatment (Figure 6a–c). In contrast, in guts dissected from larvae treated with 250 µg/µL crude leaf extract or 250 µg/µL of the three phytoecdysteroids (20-hydroxyecdysone, makisterone A and cyasterone) isolated from the leaf extract, the actin filaments were smaller and their amount reduced (Figure 6d–i). The damage observed in these experiments continued until the insects died. Overall, larvae exposed to crude leaf extract or its phytoecdysteroid fraction had less actin fibers and smaller, abnormally shaped nuclei. 

### 3.5. Pupation of S. littoralis following Biological Activity Treatments

**Figure 7:**
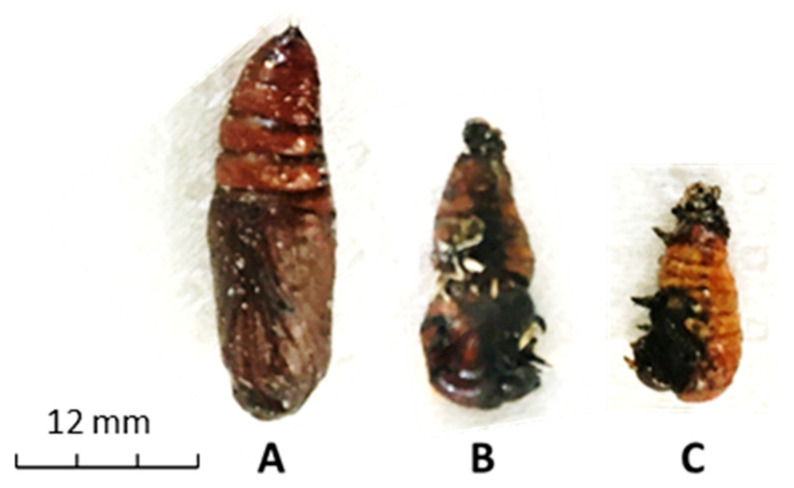
Metamorphosis of control and treated *S. littoralis*. Pupation of *S. littoralis* after 15 days of exposure (feeding for 4 days) on *A. iva* crude leaf extract. Control (treated with water) (**A**), 250 µg/µL *A. iva* crude leaf extract (**B**) and 250 µg/µL of three fractionated and purified phytoecdysteroids from *A. iva* leaf extract fractions (20-hydroxyecdysone, makisterone A and cyasterone) (**C**). Deficient development of pupation in (B) and (C) is due to lower levels of the ecdysteroids responsible for molting.

Larvae fed 250 µg/µL of crude leaf extract or 250 µg/µL of its phytoecdysteroid fraction for 8 days were unable to complete their development and pupate after 15 days (Figure 7). Figure 7b,c shows the incomplete pupae obtained; the dying larvae had short limbs, small heads, decreased weight, and only the stomach and chest pupated, which was considered non-pupation. None of them completed their development to adult moths. 

## 4. Discussion 

As predicted by Taha-Salaim et al. [26], Israeli *A. iva* crude leaf extract and its fractionated phytoecdysteroids (20-hydroxyecdysone, makisterone A and cyasterone) significantly reduced the development and survival of *S. littoralis*. These effects were pronounced throughout all larval developmental stages, including pupation. It has been shown that phytoecdysteroids negatively affect lepidopteran pests, whereas other insect species tolerate them [26]. We found that *S. littoralis* first- and third-instar larvae fed on *A. iva* crude leaf extract (50, 100 and 250 µg/µL) for 4 and 8 days, respectively, had increased mortality, reduced LWG and decreased RGR compared to the control treatment. Similarly, phytoecdysteroids from *A. iva* have been found to reduce the fertility and fecundity of *Bemisia tabaci* and *Oligonychus perseae* [29], albeit at different concentrations from those used here with *S. littoralis*, due to the difficulty in controlling *S. littoralis*, especially in advanced larval stages [36]. Intensive application of broad-spectrum insecticides has given rise to *S. littoralis* populations that are resistant to organophosphate, carbamate and pyrethroid pesticides. Moreover, commercial insecticides based on *Bacillus thuringiensis* fail to adequately control *S. littoralis* [37]. *S. littoralis* nucleopolyhedrovirus (NPV) has been intensively studied as a biopesticide for use in cotton and vegetable crops [38]. Reduced egg viability was reported for *S. littoralis* treated with sublethal doses of NPV, but no effect was found on male or female reproductive systems [39]. 

Our results are in agreement with previous studies suggesting that insect herbivores cannot develop and survive when fed on phytoecdysteroid-treated leaves. Ecdysteroids inhibited feeding of *Pieris brassicae* and *Mamestra brassicae* larvae when given at 200 mg/kg fresh weight in sucrose solution [40]. Exogenous application of ecdysteroids was shown to be lethal to *Plodia interpunctella* and *Bombyx mori* larvae; ingestion of these compounds was toxic to the midgut epithelial cells [41,42,43]. In our study, *A. iva* crude leaf extract was most effective at the highest concentration applied, indicating a dose-dependent effect, in agreement with other studies [44]. 

In our study, LWG and RGR of *S. littoralis* were affected by feeding on 250 µg/µL methanolic crude leaf extract dissolved in ddH_2_O, regardless of larval age, in agreement with recent research using a methanolic extract of *Ajuga remota* leaves containing cyasterone and ecdysterone, which disrupted the molting cycle in *Bombyx mori* and *Spodoptera frugiperda* [45]. Moreover, Slama et al. [46] found that cyasterone and turkesterone are the most effective lepidopteran- and coleopteran-specific ecdysteroids. 

In the current study, we did not fractionate clerodanes from *A. iva* crude leaf extract due to the difficulty involved in calibrating the protocol for fractionation, and to the high cost of commercial clerodane standards. Kubo [47] conducted an artificial diet-feeding assay with the wheat aphid *Schizaphis graminum* and showed that ajugasterone C (a clerodane) was 10-fold more potent as a feeding deterrent than 20-hydroxyecdysone, and 30-fold more potent than cyasterone [47]. 

In our study, we only used a few commercial standards because they are very expensive and are not feasible as a control treatment. We could not use them at the same concentrations as the applied treatment. Therefore, we conducted an experiment with a mixture of three commercial standards (ecdysterone, makisterone A and cyasterone) at a maximum concentration of 100 ppm each, which is very low compared to the concentration in the crude leaf extract and phytoecdysteroid fraction treatments (250,000 ppm). *S. littoralis* were fed on castor bean leaf treated with 100 ppm of the mixture for 8 days. No significant effect of the mixture on *S. littoralis* was seen. Tanaka [48] reported altered epidermal sensitivity to 20-hydroxyecdysone at 300 ppm ecdysone, higher than the standard concentration used in our study. 

In the present study, we observed suppressed pupation of *S. littoralis* due to the reduction in LWG; the larvae did not reach the threshold weight for pupation and they died because they could not complete their life cycle. Histological observations of the gut showed that *S. littoralis* is very sensitive to *A. iva* crude leaf extract and the mixture of the three fractionated phytoecdysteroids (20-hydroxyecdysone, makisterone A and cyasterone). The larval gut cells showed histolysis with clear signs of apoptosis. The gut epithelium showed massive deterioration; there was destruction of the microvilli of the columnar cells, and formation of vacuoles. In smaller larvae, mortality occurred during molting between instars, whereas in bigger larvae, most mortality was at the prepupal stage. Our results of gut cell destruction support the notion that the effect on pupation could be a consequence of disruptions in hormonal balance effected by internal levels of ecdysone. External ecdysteroid detoxification is one of the main ways in which insects overcome the toxic effects of these compounds. The transition from one stage in ovarian development to another, such as from previtellogenesis to vitellogenesis and then chorionogenesis, is governed by the actions of several pathways that respond to different titers of 20-hydroxyecdysone [49].

Feeding on phytoecdysteroids such as ecdysterone, polypodine B and ponasterone A induces ecdysial failure associated with the appearance of larvae having two head capsules and developmental anomalies during metamorphosis in *Acrolepiopsis* *assectella* [50]. Because the reduced growth rate (Figure 3 and Figure 4) suggests that larvae are adversely affected by ingestion of the crude leaf extract and of the mixture of three phytoecdysteroids from *A. iva,* we assume that the effect observed on LWG and RGR reflects another possible mode of action of phytoecdysteroids. Abnormal gut development can lead to reduced LWG, leading to mortality. In the present study, disruptions in *S. littoralis* gut morphology and disappearance of microfilament structures in actin (Figure 6) could be a consequence of the phytoecdysteroid titers in the *A. iva* crude leaf extract. Actin microfilaments in particular have been associated with the rounding and loss of adhesion that frequently occur with viral infection or transformation in response to secondary metabolites [51,52], with the intracellular transport of viral structural proteins and viral particles [53], with the budding process of many enveloped viruses [54], and with the assembly of virions in the cytoplasm [55] and in the nucleus [56]. 

## 5. Conclusions

Our data suggest that the phytoecdysteroids and clerodanes of *A. iva* may be useful for the management of economically important insect pests such as *S. littoralis*, while reducing the risks to human health and the environment.
